# Supplementary material for: The global apparel industry is a significant yet overlooked source of plastic leakage
Source: Nat Commun. 2024 Jun 12;15:5022. doi: 10.1038/s41467-024-49441-4 (PMC11169549; doi:10.1038/s41467-024-49441-4)
Supplement: Supplementary file 1 — Supplementary Information [file 41467_2024_49441_MOESM1_ESM.pdf]

Supplementary Material for :

The global apparel industry is a significant yet overlooked source of plastic leakage

Author list: Anna Kounina[1*†], Jesse Daystar[2,3†], Sophie Chalumeau[1], Jon Devine[2], Roland Geyer[4], Steven Pires[2], Shreya Uday Sonar[5], Richard A.Venditti[6], Julien Boucher[7]

† these authors contributed equally to this work

Affiliations

[1]Quantis Switzerland, Rue de la Gare de Triage 5, 1020 Renens, Switzerland
[2]Cotton Incorporated, 6399, Weston Parkway, Cary, NC 27513, United States
[3]Nicholas School of the Environment, Duke University, Durham, NC 27708, United States
[4]Bren School of Environmental Science and Management, University of California, Santa Barbara, CA 93106, United States
[5]Quantis United States, 66 Long Wharf, 2-West Boston, MA 02110, United States
[6]Department of Forest Biomaterials, College of Natural Resources, North Carolina State University, Raleigh, NC 27695, United States.
[7]EA - Earth Action, Chemin des vignes d'argent 7, 1004 Lausanne, Switzerland

*Corresponding author. Email: Kounina.anna@quantis.com

This PDF file includes:

Additional description of the methods

Supplementary Figures and Tables:

- Supplementary Table 1. Consumption numbers by market and fiber type, in Mt per year
- Supplementary Table 2. Gross domestic product per capita, in purchasing power parity, international dollars
- Supplementary Fig. 1: Plastic leakage across the life cycle of cotton apparel
- Supplementary Fig. 2: Plastic leakage across the life cycle of polyester apparel
- Supplementary Fig. 3: Plastic leakage across the life cycle of apparel from other fibers
- Supplementary Fig. 4: Geographies assessed along the value chain, from pellets production to second life
- Supplementary Table 3: Apparel manufacturing by country - Synthetic apparel
- Supplementary Table 4: Apparel manufacturing by country - Cotton apparel
- Supplementary Table 5: Countries of export of used textile for the EU-28 high income countries
- Supplementary Table 6: Countries of export of used textile for the EU-28 low income countries
- Supplementary Table 7: Countries of export of used textile for the US
- Supplementary Table 8: Countries of export of used textile for Japan
- Supplementary Table 9: Countries of export of used textile for China
- Supplementary Table 10: Countries of export of used textile for India
- Supplementary Table 11: Countries of export of used textile for Brazil
- Supplementary Table 12: Summary and data quality assessment for background data provided in the PLP methodology
- Supplementary Fig. 5: Textile-specific MWI calculation methodology
- Supplementary Table 13: MWI refined for textile
- Supplementary Table 14: MWI uncertainty intervals for textile
- Supplementary Table 15: MWI values for textile from different methodologies
- Supplementary Table 16: MWI by groups of countries
- Supplementary Table 17. Key results of apparel consumption, plastic waste and leakage per life cycle stage and primary market for cotton and synthetic fibers, in Mt per year.

Methods

ESTIMATION OF CONSUMPTION PER MARKET

There is no peer-reviewed publication that contains estimates for end-use apparel consumption by fiber type and region. This research introduces figures for apparel consumption in the US, the EU-28 High and low income countries, China, India, Japan, Brazil, and the aggregated Rest of the World.

Apparel consumption data are for the 2019 calendar year or the 2018/19 crop year, which is the latest calendar or crop year before the distortive effects of the COVID pandemic.

Cotton apparel

For cotton apparel, estimates were generated using an apparent consumption approach. The starting point for these calculations was the quantity of cotton fiber consumed by spinning mills (e.g., yarn production) for each primary market or country *c* [1]. To these volumes, imports were added and exports were subtracted at each downstream stage of the value chain (fabric manufacturing and cut and sew assembly into finished consumer products) [2, 40]. Trade volumes were adjusted to account for blending and loss throughout the value chain using the trade category specific conversion factors (i) established by the United States Department of Agriculture (USDA) [3].

$$\mathbf{CottonFiberCons_c = DomesticCottonMillUse_c + (Yarn\ Imports_i * Conversion\ Factor_i) - (Yarn\ Exports * Conversion\ Factor_i) + (Fabric\ Imports_i * Conversion\ Factor_i) - (Fabric\ Exports_i * Conversion\ Factor_i) + (Finished\ Textile\ Imports_i * Conversion\ Factor_i) - (Finished\ Textile\ Exports_i * Conversion\ Factor_i)} \tag{1}$$

A result of these calculations was a total volume of cotton available for consumers. These figures needed to be scaled down to isolate apparel (i.e., exclude fibers meant for other uses such as non-wovens or home furnishings). Several markets (US, EU-28 high and low income, and Japan) are dominated by imports. Imports are classified according to end-use (e.g., apparel and home furnishings), and the proportion of apparel relative to other end-uses was derived for each import-dominant market. These proportions were applied against the result from the apparent consumption process to establish estimates for cotton consumed in apparel in import-dominant markets.

$$\mathbf{CottonApparelCons_{c\ imp} = CottonFiberCons_{c\ imp} * CottonApparelProportion_{c\ imp}}$$

† c imp denotes import-dominant apparel markets (2)

For China, India, and Brazil, domestic apparel manufacturing represents a larger share of apparel available for consumers, and trade data could not be leveraged in the same way to deflate total cotton end-use to apparel end-use. However, the proportion of apparel relative to other end-uses was consistent across import-dominant regions (between 75% and 78%). Given the international consistency, this average was applied to deflate apparent consumption results for total cotton end-use for China, India, and Brazil to estimates for the volume of cotton devoted to apparel in those markets.

$$\mathbf{CottonApparelCons_{c\ mfg} = CottonFiberCons_{c\ mfg} * ApparelProportion_{c\ mfg}}$$

† c mfg denotes markets with significant domestic apparel manufacturing (3)

For the Rest of the World, cotton world production numbers from the USDA are taken and multiplied by 75% to isolate apparel, using apparel's estimated share of end-use consumption from the markets of focus. The quantity of cotton apparel in the specific markets studied is then subtracted, to obtain the quantity for the Rest of the World.

$$\mathbf{CottonApparelCons_{Global}} = \mathbf{CottonMillUse_{Global}} * \mathbf{75\%}$$

$$\mathbf{CottonApparelCons_{RoW}} = \mathbf{CottonApparelCons_{Global}} - \sum_{\mathbf{c}} \mathbf{CottonApparelCons_{c}} \quad (4)$$

<u>Man-made apparel</u>

The apparent consumption approach could not be applied for man-made apparel. There are two reasons. One is because the geographies for published man-made fiber production and consumption data are often by region rather than by country. This prevents the alignment of trade data with yarn production data. Another obstacle for apparent consumption methods and apparel derived from man-made fibers is that there is a greater diversity of end-uses for man-made fibers (e.g., automobile upholstery), and there is no known way to comprehensively estimate the volumes devoted to these alternate end-uses. For the import-dominant markets (US, EU-28, and Japan), imports were assumed to represent apparel constructed with man-made fibers (i.e., petroleum-derived synthetic fibers and cellulosic-derived fibers) consumption. Imported apparel weight volumes [2,40] were multiplied by USDA conversions factors [4] to incorporate fiber loss and blending along the value chain. In a robustness check, apparel import figures for cotton were compared against those derived under the apparent consumption approach. The values closely resembled each other, suggesting that the use of purely import-based estimates is a valid alternative to apparent consumption for apparel composed of man-made fibers for these markets.

$$\mathbf{ManmadeApparelCons_{c\ imp}} = \mathbf{ManmadeApparelImports_{c\ imp}} \quad (5)$$

There is more domestic apparel manufacturing in Brazil, China, and India, and apparel import data cannot be considered representative of consumers' use. A solution was to use cotton's share of apparel. Cotton's share of apparel was relatively consistent across import markets (EU, US and Japan), ranging between 45.5% and 56.7%. It was assumed that cotton's share of apparel could be similar in Brazil, China, and India. Specifically, it is assumed to be 50% for Brazil and India. For China, where man-made fiber production is high, we considered a range for cotton's share of apparel between 40% and 50%.

$$\mathbf{ManmadeApparelCons_{Brazil}} = \mathbf{CottonApparelCons_{Brazil}} \quad (6)$$

$$\mathbf{ManmadeApparelCons_{India}} = \mathbf{CottonApparelCons_{India}} \quad (7)$$

$$\mathbf{ManmadeApparelCons_{China-Min}} = \mathbf{CottonApparelCons_{China}} \quad (8)$$

$$\mathbf{ManmadeApparelCons_{China-Max}} = \frac{\mathbf{60\%}}{\mathbf{40\%}} * \mathbf{CottonApparelCons_{China}} \quad (9)$$

For the Rest of the World, the assumption that 75% of global cotton use is devoted to apparel was leveraged. It was then assumed that cotton had a 50% market share of apparel. The result is that the estimated volume of synthetic and cellulosic fibers derived fibers flowing into apparel is the same as cotton.

$$\mathbf{ManmadeApparelCons_{RoW}} = \mathbf{CottonApparelCons_{RoW}} \quad (10)$$

Due to differences in the implications for plastic leakage for synthetic (e.g., polyester) and cellulosic-derived (i.e., viscose) man-made fibers, estimates for man-made fibers were

separated according to source material. Published figures for the share of petroleum-derived and cellulosic-derived fiber in apparel are not available. To approximate these volumes, figures for global textile fiber production [5] were assumed consistent across markets. Specifically, 91% of man-made fibers were considered synthetic and 9% considered cellulosic-derived.

$$\mathbf{SyntheticApparelCons_c} = \mathbf{ShareofSyntheticInManmade_c} * \mathbf{ManmadeApparelCons_c} \quad (11)$$

<u>Non-cotton natural fiber apparel</u>

Estimates for yarn production for non-cotton natural fibers (wool, linen, silk) are not available. This prevents the use of apparent consumption for describing apparel consumption using net apparel consumption methods. At the most granular internationally shared level (HS6), import classifications prevent representative estimates from being derived for apparel made from non-cotton natural fibers. However, the most detailed US import categories (HS10) enable further delineation by fiber content. These data show non-cotton natural fibers represent 4% of US apparel imports, and a 4% share for non-cotton natural fibers is assumed for all other markets.

The consumption numbers are summarized in Supplementary Table 1.

Supplementary Table 1. Consumption numbers by market and fiber type, in Mt per year

	US	EU - high income	EU - low income	JP	BZ	IN	CN	RoW	Total
Cotton	2.50	1.78	0.69	0.45	0.56	2.03	2.3	4.4	15
Synthetic - mean	2.20	1.92	0.63	0.55	0.51	1.9	2.5	4.4	15
Synthetic - low							2.1	4.2	14
Synthetic - high							3.0	4.6	15
Other fibers	0.41	0.34	0.12	0.09	0.09	0.34	0.4	0.78	2.6
Total - mean	5.11	4.03	1.44	1.10	1.16	4.2	5.2	9.6	32
Total - low							4.8	9.4	31
Total - high							5.7	9.8	33

A double check is performed to make sure that these numbers are consistent with the economic reality, and in particular the quantity of apparel consumed in the Rest of the World compared to the 7 regions singled out. For this, data from the IMF [6] on purchasing price parity (PPP) per capita and economic activity are compared. Supplementary Table 2 shows that the PPP per capita of the 164 countries in the set "Emerging and Developing Economies" is about a quarter of that from the EU.
Moreover, Ward and Neumann[7] shows that lower income brackets spend as much as 40% of their income on food alone. And even when incomes increase to $10k per capita, the volume spent on clothes is about one third of the amount in markets with average income. This indicates that clothing spending is exponential with income, and while there may be many more consumers at the lower income levels, it takes many of them to equal spending in developed markets. In addition, lower income markets will be recipients of used apparel from developed markets, which will also weigh on their consumption of new apparel. This suggests that significant proportion of their limited spending could go to used items rather than new ones.

Supplementary Table 2. Gross domestic product per capita, in purchasing power parity, international dollars

	2015	2016	2017	2018	2019	2020	2021	2022
European Union	41'225	41'994	43'185	44'058	44'845	42'224	44'514	45'857
Emerging market and developing economies	10'164	10'460	10'809	11'166	11'423	11'058	11'705	12'016
Ratio EU/Developing - per capita	4.1	4.0	4.0	3.9	3.9	3.8	3.8	3.8

PLASTIC FLOWS ALONG THE APPAREL VALUE CHAIN

The assessment follows a lifecycle thinking by considering all identifiable activities on the value chain and upstream (i.e., the full range of activities needed to create, use and dispose of the apparel), in order to provide as comprehensive a view as possible of the product's cradle-to-grave life cycle. Supplementary Fig. 1, 2 and 3 show the plastic flows taken into account at each stage of the apparel value chain, for cotton, synthetic and other fibers apparel respectively.

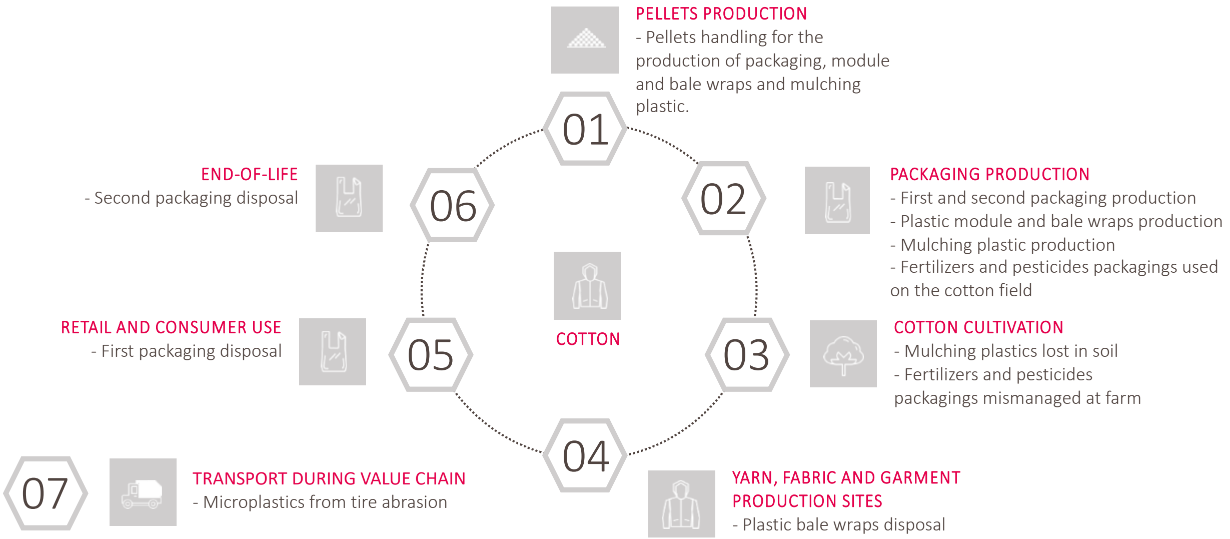

Supplementary Fig. 1: Plastic leakage across the life cycle of cotton apparel

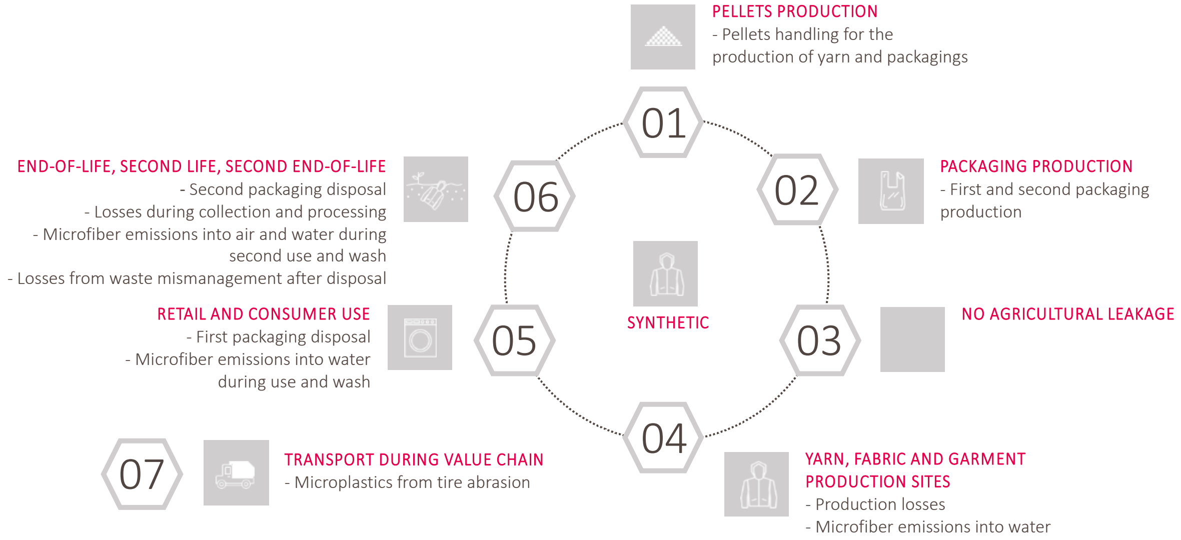

Supplementary Fig. 2: Plastic leakage across the life cycle of synthetic apparel

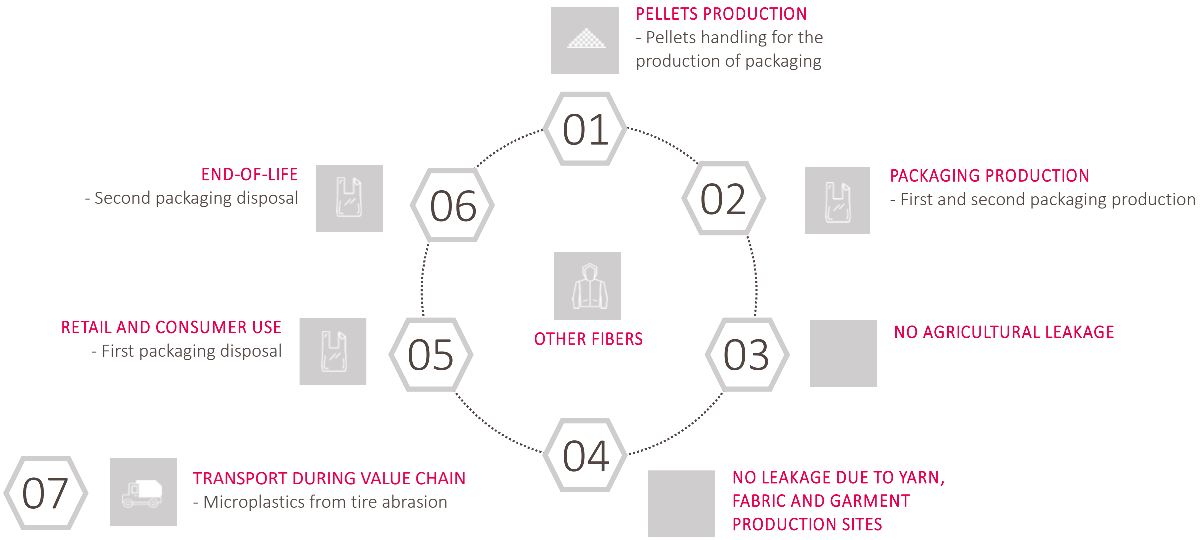

Supplementary Fig. 3: Plastic leakage across the life cycle of apparel from other fibers

Details on the plastic flows presented in Supplementary Fig. 1, 2 and 3 for each life cycle stage are given below:

1. Pellet production: This includes the pellet production stage to produce plastic materials such as polyester yarn and plastic packaging.
2. Plastic formation: This includes production of the plastic material such as polyester fibers, packaging, and module wraps from plastic pellets.
3. Agricultural plastics: This includes losses from plastic applied to agricultural soil, such as mulching plastic, and items which may be mismanaged at the farm level such as module wraps or crop input packaging.
4. Yarn, fabric and apparel production sites: This includes any losses during the apparel manufacturing stage including producing yarn or fabric, wet processing, and apparel assembly.
5. Retail and consumer use: This stage covers plastic leakage from disposal of the primary packaging, and microfiber emissions to water and soil from the apparel during washing.
6. End-of-life in the first market, recover for reuse and end-of-life in the second market: This life cycle stage represents the appropriate end-of-life pathway for apparel in each country of consumption. The shares of items landfilled, incinerated, recycled, exported for second life and the use pattern in second life are incorporated.
7. Transport throughout the value chain: This covers microplastic losses from truck tire abrasion that occurs across the value chain.

GEOGRAPHIES ALONG THE APPAREL VALUE CHAIN

Some processes within the system boundaries might take place anywhere or within multiple life cycle stages, such as the pellet and packaging production, respectively. In contrast, some have definite geographies, with specific variations in plastic flows associated with said geographies. The different geographies considered at each stage are summarized in Supplementary Fig. 4.

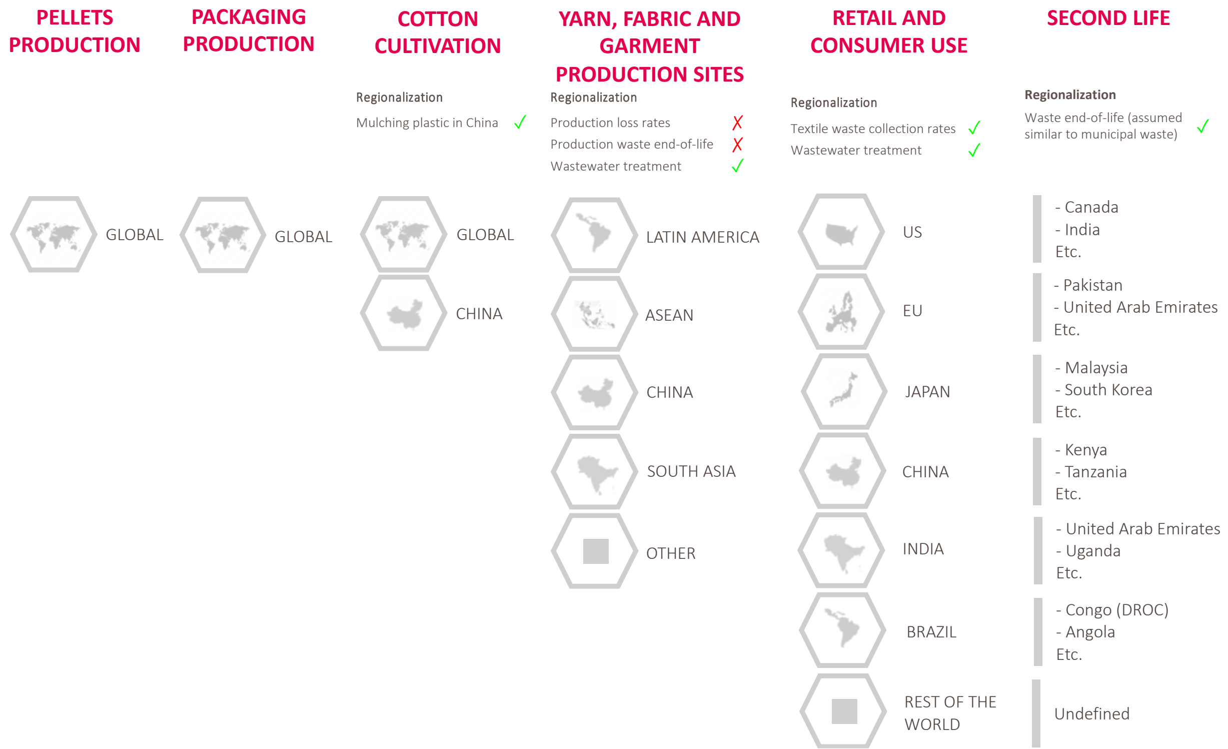

Supplementary Fig. 4: Geographies assessed along the value chain, from pellets production to second life

For apparel second life, only the main export countries are given in Supplementary Fig. 4, and the complete lists of export markets are given in Supplementary Tables 6 to 12.

For cotton cultivation, China was treated differently as the plastic flows associated with the cultivation processes differ between China and elsewhere in the world.

The location specific textile production shares related to different sales markets were extracted from Trade Data Monitor [2] and are summed in Supplementary Tables 3 and 4.

Supplementary Table 3: Apparel manufacturing by country - Synthetic apparel

		Primary sales markets considered					
		EU-28 High and low income	US	Japan	China	India	Brazil
Manufacturing countries (2)	Latin America	1%	28%	0%	0%	0%	0%
	ASEAN (Association of Southeast Asian Nations)	19%	25%	29%	0%	0%	0%
	South Asia	26%	6%	6%	0%	0%	0%
	China	39%	33%	63%	100%	0%	0%
	India	0%	0%	0%	0%	100%	0%
	Brazil	0%	0%	0%	0%	0%	100%
	Undefined	16%	9%	1%	0%	0%	0%

Supplementary Table 4: Apparel manufacturing by country - Cotton apparel

		Primary sales markets considered					
		EU-28 High and low income	US	Japan	China	India	Brazil
Manufacturing countries	Latin America	1%	28%	1%	0%	0%	0%
	ASEAN	8%	19%	35%	0%	0%	0%
	South Asia	59%	25%	15%	0%	0%	0%
	China	13%	21%	46%	100%	0%	0%
	India	0%	0%	0%	0%	100%	0%
	Brazil	0%	0%	0%	0%	0%	100%
	Undefined	20%	7%	2%	0%	0%	0%

For the EU-28, the US and Japan, we based the production mix on trade data [2].

For China, India and Brazil, since they are major production countries, it is not possible to rely on trade data, and we assumed that the total apparel consumption comes from domestic production.

At the end-of-life of apparel, some is recycled, landfilled or incinerated within the same country, whereas some is exported to other countries to be reused and eventually disposed. We extracted the share of textiles exported at the end-of-life (trade code HS 6309) over total textile consumption in the market from trade data [2]. We assume that the same percentage of used exports applies to apparel as to other textiles. The quantities are reported in Supplementary Tables 5 to 11.

Supplementary Table 5: Used apparel exported by the EU-28 High income countries

Export country	Mass exported (in Mt)
Pakistan	11%
Tunisia	7.3%
United Arab Emirates	7.2%
Ghana	5.8%
Cameroon	4.0%
Türkiye	3.9%
Ukraine	3.2%
India	2.6%
Nigeria	2.4%
Togo	2.1%
Russian Federation	1.4%
Guinea	1.4%
Benin	1.4%
Kenya	1.3%
Belarus	1.2%
Senegal	1.1%
Oman	0.81%
Côte d'Ivoire	0.72%
Morocco	0.68%
Chile	0.64%
Madagascar	0.63%
Angola	0.62%
United Kingdom	0.60%
Dem. Rep. of the Congo	0.60%
Niger	0.58%
Congo	0.58%
South Africa	0.55%
Burkina Faso	0.54%
Mali	0.53%
Albania	0.51%
Haiti	0.48%
Gabon	0.47%

Iraq	0.46%
Jordan	0.43%

Supplementary Table 6: Used apparel exported by the EU-28 Low income countries

Export country	Mass exported (in Mt)
Pakistan	18%
Ukraine	13%
United Arab Emirates	7.5%
Togo	7.4%
Benin	5.2%
Russian Federation	4.1%
Belarus	3.8%
Ghana	3.4%
Nigeria	3.2%
India	2.8%
Kenya	2.7%
Niger	2.1%
Cameroon	2.1%
Djibouti	2.0%
Türkiye	1.7%
United Rep. of Tanzania	1.7%
Uganda	1.7%
Mali	1.3%
Guinea	1.2%
Congo	0.91%
Angola	0.90%
Mozambique	0.88%
Georgia	0.81%
Côte d'Ivoire	0.68%
Tunisia	0.64%
Morocco	0.62%
Malawi	0.61%
Zambia	0.58%
Senegal	0.54%
Chile	0.54%
Serbia	0.53%
Burundi	0.50%

Gambia	0.50%

Supplementary Table 7: Used apparel exported by the US

Export country	Mass exported (in Mt)
Canada	16%
India	13%
Guatemala	12%
Chile	8%
Honduras	7%
Dominican Republic	6%
Pakistan	5%
United Arab Emirates	4%
Nicaragua	4%
Mexico	3%
El Salvador	3%
Congo (DROC)	2%
Philippines	2%
Malaysia	2%
Liberia	1%
Kenya	1%
Angola	1%
Tunisia	1%
Tanzania	1%
Senegal	1%
Costa Rica	1%
Guinea	1%
Ukraine	1%

Supplementary Table 8: Used apparel exported by Japan

Export country	Mass exported (in Mt)
Malaysia	52%
South Korea	17%
Philippines	15%
Cambodia	4%
Pakistan	3%
India	3%
Hong Kong	3%
Thailand	2%

Supplementary Table 9: Used apparel exported by China

Export country	Mass exported (in Mt)
Kenya	20%
Tanzania	8%
Angola	8%
Pakistan	7%
Philippines	7%
Nigeria	6%
Mozambique	5%
Ghana	4%
Madagascar	3%
Haiti	2%
Uganda	2%
India	2%
Cambodia	2%
Cameroon	2%
Turkey	2%
Guinea	2%
Thailand	2%
Cote d'Ivoire	2%
Benin	1%
Malawi	1%
United Arab Emirates	1%
Congo (DROC)	1%
Iraq	1%
Togo	1%
Rwanda	1%
Singapore	1%

Supplementary Table 10: Used apparel exported by India

Export country	Mass exported (in Mt)
United Arab Emirates	25%
Uganda	11%
Kenya	11%
Congo (DROC)	8%
Tanzania	5%
Thailand	5%
Philippines	4%
Malaysia	3%
Malawi	3%
Angola	3%
Mozambique	3%
Ghana	2%
Zambia	2%
Chile	1%
Rwanda	1%
Madagascar	1%
Congo (ROC)	1%
Jordan	1%
Guinea	1%
Burundi	1%
Sudan	1%
Japan	1%

Supplementary Table 11: Used apparel exported by Brazil

Export country	Mass exported (in Mt)
Congo (DROC)	52%
Angola	29%

PLASTIC LEAKAGE ALONG THE APPAREL THE VALUE CHAIN

Plastic leakage is defined as the mass of plastic leaving the human environment and terminating in the natural environment. Leakage is a result of both loss and release through transfer and redistribution pathways. Losses according to Peano et al. [8] are the mass of plastic that leaves a managed product or waste management system. All losses do not necessarily end up in the natural environment. For example, part of the microfiber losses through apparel washing are recaptured in wastewater treatment plants. The fraction that then leaves the human environment for the natural environment is said to be released. The sum of the plastic released into different environmental compartments corresponds to the total leakage.

At each step of the apparel value chain, we assessed the macro- and micro-plastics leakage by combining the plastic flows with the loss and release rates provided by Peano et al. [8]. These include pellet losses during manufacturing, microplastic fibers losses through laundering, microplastic release through wastewater, macroplastic losses during waste disposal, and microplastic losses and release through transport. Supplementary Table 12 shows the different plastic leakage routes for each stage of the life cycle of a textile product, and summarizes the related background data that are further detailed in Peano et al. [8], as well as the estimated data quality.

Mismanagement waste indexes (MWI) in particular are a key factor influencing the results of this study. The MWI used in this study were compiled by Peano et al. [8], and account for both uncollected and poorly managed waste. This follows the definition of mismanaged waste from Jambeck et al. [10] as “material that is either littered or inadequately disposed. Inadequately disposed waste is not formally managed and includes disposal in dumps or open, uncontrolled landfills, where it is not fully contained”. Poorly managed waste according to Peano et al. [8] includes:

- Dumping: In low-income countries, collected waste can end up in an open dump, which is prone to pollute nearby aquifers, water bodies and settlements.
- Non-sanitary landfills: In transition countries, landfills planned as controlled engineered sites can end up being mismanaged (e.g., light plastic waste may be blown away by wind, or carried away by runoff) (Velis et al. 2017).

Supplementary Table 12: Summary and data quality assessment for background data provided in the plastic leakage methodology

Life cycle stages	Nature of plastic leakage	Key background data	Data quality
Suppliers	Microplastics from pellet production	Pellet loss rates:<br>Lassen et al. (2015) [11], Sundt et al. (2014) [12], Cole and Sherrington (2016) [13]	Low
Production (company-owned)	Macroplastics from product and packaging production	Mismanaged waste rates per country:<br>Kaza et al. (2018) [14]	Low
Product use	Microfiber from textile washing and wearing	Release rate after wastewater treatment:<br>Country data compiled based on various sources (Van Drecht et al. 2009 [15]; Williams et al. 2012 [16]; Baum et al. 2013 [17])	Average
Product end-of-life	Macroplastics from textile products end-of-life (incineration, landfilling, recycling, reuse) in hotspot countries	Mismanaged waste indexes (MWI) for textile, for hotspot countries country: see next section	Average
	Macroplastics from textile products end-of-life in other countries, and from packaging end-of-life in all countries	Mismanaged waste indexes (MWI) per country:<br>Kaza et al. (2018) [14]	Low
Transport	Microplastics from tire abrasion	Unice et al. (2018) [18]<br>Hann et al. (2018) [19]	High

*For the countries identified as key leakage hotspots, individually refined MWI for textiles specifically were compiled (see the next section) rather than using the data from Kaza et al (2018).

MISMANAGED WASTE INDEXES (MWI) FOR TEXTILE WASTE IN HOTSPOT COUNTRIES

For the countries identified as apparel leakage hotspots (the US, the EU, China, India, Pakistan and Brazil), we refined the MWI to represent textile waste specifically.

The methodology we adopted to obtain a textile-specific MWI value in these countries can be divided into three steps:

1. Collect country-specific data on separate collection rate for textile and on end-of-life for the separately collected textiles.
2. Collect data on collection rate and end-of-life of municipal solid waste.
3. Combine information from previous steps to derive the value of MWI for textile waste in the country. More specifically, separately collected textiles will follow e-o-l paths specific for them, whereas the rest will be disposed as municipal solid waste.

Supplementary Fig. 5: Textile-specific MWI calculation methodology

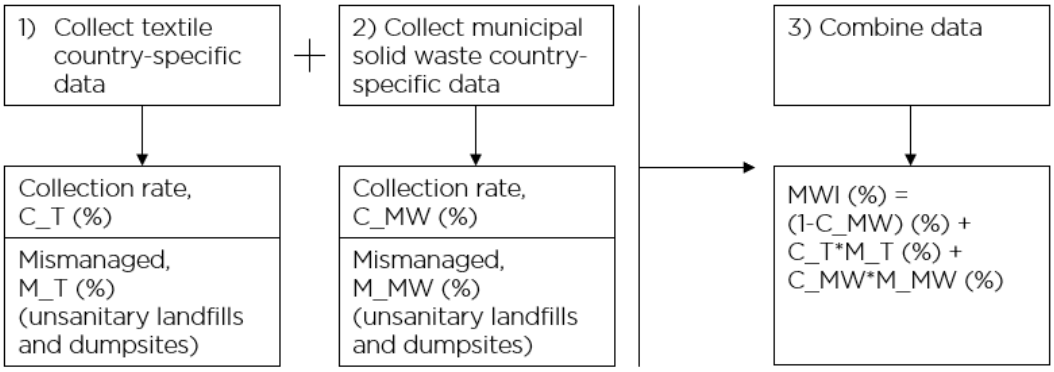

The first step, aiming at determining a separate collection rate for textile, and fate of these textiles, is based on a thorough literature review. Given the limited availability of knowledge on this subject, there is a lack of comprehensive and consistent data. In the following sections, we provide an overview of the data collected for each country included in our study and explain how we used them to derive textile specific MWIs.

- For the US, data on textile collection rate were found in [20,21] and data on their fates were found in [21].
- For China, data on textile collection rate and fates were both found in [22]. There was no reference to any kind of loss rates in collecting and sorting, nor any disposal of collected textiles. A value of 20% was applied, in line with the percentage of losses in the collection and recycling process.
- For India, data were found in [23, 24] and in its technical appendix.
- For Brazil, data on textile collection rate and its fates were found in [25].
- For Pakistan, data were found in [26] and [27]. There was no mention of any kind of separate collection for textile, leading us to assign a collection rate of 0%. Both sources confirm that recycling facilities in Pakistan primarily handle imported textile waste rather than domestically generated waste.
- For Japan, data were found in [28].

To date, reporting practices on the recovery of textile waste are not yet harmonised across the board and authors expect that more accurate data will be available in the future.
The second step relies on a review of the most recent data on municipal waste management in the country. It should cover the collection rate, as well as the shares of management of collected municipal waste between incineration, sanitary landfill, non-sanitary landfill and dumpsite. Quantitative data were found for all countries in [29,30,33,34].
The third and last step consists in combining data that are textile-specific with data on municipal waste management.
We multiply the textile collection rate with the different fates' shares, to determine the e-o-l of the separately collected portion of textile. The possible e-o-l are recycling, reuse, export, incineration, sanitary landfill, non-sanitary landfill and dumpsite. It is important to note that even textile waste that has been separately collected can still contribute to the MWI, if it ends in non-sanitary landfills or dumpsites.
We proceed analogously for the portion of textile that ends up in the municipal solid waste, and for which the possible e-o-l are incineration, sanitary landfill, non-sanitary landfill and dumpsite.
The MWI for textile waste is eventually computed as follows:

$$\begin{aligned}\mathbf{MWI_{textile}}(\%) = {} & \mathbf{Uncollected\ textile\ waste}\ (\%) + \\ & \mathbf{separately\ collected\ textile\ waste\ in\ non\ sanitary\ landfills}\ (\%) + \\ & \mathbf{textile\ waste\ in\ MSW\ in\ non\ sanitary\ landfills}\ (\%) + \\ & \mathbf{separately\ collected\ textile\ waste\ in\ dumpsites}\ (\%) + \\ & \mathbf{textile\ waste\ in\ MSW\ in\ dumpsites}\ (\%) \end{aligned} \quad (12)$$

- For the EU28 high income and low group, the approach adopted was slightly more complex. For the high GDP group, data on six countries were found in [31]. The population of these six countries represent around 55% of the whole group. The data we found included textile collection rate and fates of this textile, such as reuse, recycle, disposal (incineration, landfill and unsanitary landfill) and export. For some of these countries, the fate of exported waste was already included in the final rates given. Since this is studied separately in this article the rates for waste that was not exported were reconstructed by comparing the exported quantities with quantities found in [34] and other relevant information found in [31]. For the low income group, data on textile collection rate for four countries (Estonia, Czech Republic, Latvia and Lithuania) were found in [35], and for other two countries (Spain and Poland) in [36]. Data on the fate of the collected textile was available only for three countries (Spain, Czech Republic and Estonia). The six countries represent the 60% of the population of the group. In both cases, we calculated a weighted average value applicable to the whole group by considering the rates of all countries and factoring in their respective textile consumptions. Finally, we use the same methodology to combine data on textile waste separately collected with textile waste that ends up in municipal solid waste.

Supplementary Table 13: MWI refined for textiles

Market	MWI for textiles (%)	Reference year
EU High Income	0%	2020
EU Low Income	7.8%	2021
US	1.6%	2020
China	43%	2019
India	68%	2022
Brazil	40%	2017
Pakistan	98%	2019
Japan	1.8%	2019

UNCERTAINTY ANALYSIS

The MWI are one of the key parameters influencing the results, while exhibiting a high uncertainty due to the general lack of primary data. Therefore, we performed an uncertainty analysis on this parameter.

For the US, EU high and low income countries, China, India, Brazil, Pakistan and Japan, which have a refined MWI specific to textile, we calculated a different uncertainty range for the textile MWI and for the packaging MWI. The textile MWI uncertainty analysis is given in Supplementary Table 14.

Supplementary Table 14: MWI uncertainty intervals for textile

Market	MWI for textile – low end (%)	MWI for textile – high end (%)
EU High income	0.0%	3.8%
EU Low income	4.0%	12%
US	0.1%	3.1%
China	24%	62%
India	63%	73%
Brazil	15%	65%
Pakistan	93%	100%
Japan	0.0%	5.8%

The approach chosen to calculate this uncertainty is based on the evaluation of the standard deviation in MWI values for general waste obtained through different methodologies for the same country. The MWI values for general waste obtained through 7 different methodologies as well as the associated standard deviation for each country are provided in Supplementary Table 15.

Supplementary Table 15: MWI values for textile from different methodologies

Market	Methodologies							Standard deviation across sources
	EA – based on What a Waste 2.0 data [37]	EA – based on World Waste Status [37]	WWF ReSource 2020 [38]	Jambeck et al.2015 [10]	Lebreton & Andrady 2019 [39]	PLP 2020 [8]	PLASTEAX 2019	
EU-28	7%	15%	5%	4%	6%	11%	12%	4%
US	0%	0%	2%	2%	1%	2%	5%	2%
China	28%	85%	76%	76%	70%	71%	26%	20%
India	87%	100%	88%	88%	95%	95%	77%	5%
Brazil	35%	84%	11%	11%	42%	31%	43%	27%
Pakistan	98%	96%	87%	87%	85%	88%	92%	5%
Japan	13%	6%	2%	2%	1%	2%	5%	4%

We took the uncertainty range of the packaging MWI as the interval between the 1st and the 3rd quartile of the MWI of the economic group they belong to: High income countries (HIC), Upper middle income countries (UMC), Lower middle income countries (LMC), Low income countries (LIC). The quartiles of the MWI by economic grouping of countries is summed up in Supplementary Table 16 (classification taken from Kaza et al. [14]).

Supplementary Table 16: MWI by groups of countries

Groups of countries	1st quartile	Median	3rd quartile
HIC	0%	4.3%	23%
UMC	39%	68%	88%
LMC	79%	88%	95%
LIC	95%	95%	98%
HIC+UMC	2.7%	23%	69%
LIC+LMC	88%	95%	95%
LIC+LMC+UMC	69%	88%	95%

For the export countries, we take the uncertainty interval the 1st and 3rd quartiles of their income category. For exports for which the geography is not defined, the aggregation of the LIC, LMC and UMC is taken.

Supplementary Table 17. Key results of apparel consumption, plastic waste and leakage per life cycle stage and primary market for cotton and synthetic fibers, in Mt per year. Data for Fig. 1 and Fig 2 are expressed here. The numbers into brackets show the uncertainty intervals.

		Cotton		Synthetic		Other fibers		Total	
	Apparel consumption	15		15	[14 - 15]	2.6		32	[31 - 33]
Plastic waste	Packaging	1.7		1.7	[1.6 - 1.8]	0.31		3.8	[3.7 - 3.8]
	Garment or cotton cultivation	0.21		17	[16 - 17]	0		17	[16 - 18]
Plastic waste related to primary market	EU28 - high income	0.23		2.4		0.040		2.7	
	EU28 - low income	0.090		0.80		0.014		0.9	
	US	0.33		2.8		0.048		3.2	
	Japan	0.063		0.69		0.011		0.77	
	China	0.34		3.2	[2.6 - 3.8]	0.052		3.6	[3.0 - 4.2]
	India	0.26		2.4		0.040		2.7	
	Brazil	0.071		0.65		0.011		0.73	
	Rest of the world	0.56		5.5	[5.3 - 5.8]	0.092		6.2	[5.9 - 6.4]
Plastic leakage - per life cycle stage	First end-of-life (in primary market)	0.71	[0.44 - 1.3]	5.9	[2.9 - 8.7]	0.12	[0.08 - 0.21]	6.7	[3.4 - 10.2]
	Second end-of-life (in secondary markets)	0		1.4	[1.2 - 1.6]	0		1.4	[1.2 - 1.6]
	Others	0.055	[0.060 - 0.060]	0.14	[0.17 - 0.37]	0.0013	[0.0013 - 0.0013]	0.20	[0.23 - 0.43]
Plastic leakage - related to primary market	EU28 - high income	0.021	[0.018 - 0.15]	0.47	[0.43 - 0.82]	0.0030	[0.0006 - 0.010]	0.49	[0.44 - 1.0]
	EU28 - low income	0.0083	[0.0069 - 0.057]	0.20	[0.20 - 0.31]	0.0011	[0.0001 - 0.000]	0.2	[0.21 - 0.4]
	US	0.0092	[0.0092 - 0.084]	0.25	[0.18 - 0.38]	< 0.001		0.26	[0.20 - 0.47]
	Japan	0.0023	[0.0023 - 0.016]	0.061	[0.061 - 0.15]	< 0.001		0.064	[0.066 - 0.16]
	China	0.094	[0.094 - 0.25]	1.3	[0.71 - 2.2]	0.015	[0.021 - 0.045]	1.4	[0.85 - 2.5]
	India	0.24	[0.21 - 0.24]	1.5	[1.4 - 1.6]	0.040	[0.034 - 0.039]	1.8	[1.6 - 1.9]
	Brazil	0.025	[0.0044 - 0.044]	0.23	[0.08 - 0.38]	0.0039	[< 0.001 - 0.0072]	0.26	[0.088 - 0.43]
	Rest of the world	0.37	[0.12 - 0.51]	3.5	[1.2 - 4.9]	0.063	[0.020 - 0.088]	3.9	[1.4 - 5.5]

References

1. United States Department of Agriculture. *Production Supply and Distribution* (United States Department of Agriculture, 2019); https://apps.fas.usda.gov/psdonline/app/index.html#/app/advQuery.
2. Trade data Monitor. *Trade Data Monitor* (2020); https://tradedatamonitor.com/.
3. United States Department of Agriculture. *Raw-Fiber Equivalents of U.S. Textile Trade Data Documentation* (United States Department of Agriculture, 2019); https://www.ers.usda.gov/data-products/cotton-wool-and-textile-data/raw-fiber-equivalents-of-us-textile-trade-data-documentation.
4. United States Department of Agriculture. *Raw-Fiber Equivalents of U.S. Textile Trade Data Documentation* (United States Department of Agriculture, 2019)
5. Textile Exchange. *Preferred Fiber & Materials Market Report 2020* (Textile Exchange, 2021).
6. International Monetary Fund. *World Economic Outlook Database* (International Monetary Fund, 2022); https://www.imf.org/en/Publications/WEO/weo-database/2022/October/select-aggr-data.
7. Ward, K. & Neumann, F. Consumer in 2050. *HSBC Glob. Reseach* (2012).
8. Peano, L. et al. *Plastic Leak Project: Methodological Guidelines* (Quantis & Environmental Action, 2020).
9. Rosenbaum, R. K. et al. USEtox—the UNEP-SETAC toxicity model: recommended characterisation factors for human toxicity and freshwater ecotoxicity in life cycle impact assessment. *Int. J. Life Cycle Assess.* **13**, 532–546 (2008).
10. Jambeck, J. R. et al. Plastic waste inputs from land into the ocean. *Science* **347**, 768–771 (2015).
11. Lassen, C. et al. *Microplastics: Occurrence, effects and sources of releases to the environment in Denmark* (Danish Environmental Protection Agency, 2015); http://mst.dk/service/publikationer/publikationsarkiv/2015/nov/rapport-ommikroplast
12. Sundt, P., Schulze, P.-E. & Syversen, F. *Sources of Microplastic Pollution to the Marine Environment* (Norwegian Environment Agency, 2014).
13. Cole, G. & Sherrington, C. *Study to Quantify Pellet Emissions in the UK* (Eunomia, 2016).
14. Kaza, S., Yao, L., Bhada-Tata, P. & Van Woerdern, F. *What a Waste 2.0: A Global Snapshot of Solid Waste Management to 2050* (World Bank 2018).
15. Van Drecht, G., Bouwman, A. F., Harrison, J. & Knoop, J. M. Global nitrogen and phosphate in urban wastewater for the period 1970 to 2050. *Global Biogeochem. Cycles* **23** (2009).
16. Williams, R. et al. Assessment of current water pollution loads in Europe: estimation of gridded loads for use in global water quality models. *Hydrol. Process.* **26**, 2395–2410 (2012).
17. Baum, R., Luh, J. & Bartram, J. Sanitation: A global estimate of sewerage connections without treatment and the resulting impact on MDG progress. *Environ. Sci. Technol.* **47**, 1994–2000 (2013).
18. Unice, K., Abramson, M., Reid, R. & Panko, J. *Preliminary Tyre and Road Wear Particle Environmental Fate Assessment* (Cardno ChemRisk, 2018).

19. Hann, S. et al. *Investigating options for reducing releases in the aquatic environment of microplastics emitted by (but not intentionally added in) products* (Eunomia, 2018).

20. EPA. *Facts and Figures about Materials, Waste and Recycling. Textiles: Material-specific data* (EPA, 2023); https://www.epa.gov/facts-and-figures-about-materials-waste-and-recycling/textiles-material-specific-data

21. RRS. *Textile Recovery in the U.S., a Roadmap to Circularity* (RRS, 2020); http://recycle.com/wp-content/uploads/2020/09/2020-Textile-White-Paper-sept-15-2020.pdf

22. Spuijbroek, M. *Textile Waste in Mainland China. An Analysis of the Circular Practices of Post-Consumer Textile Waste in Mainland China* (Intern Embassy of the Kingdom of the Netherlands, 2019).

23. Fashion For Good. *Wealth in Waste, India's Potential to Bring Textile Waste Back into the Supply Chain* (Fashion For Good, 2022); https://reports.fashionforgood.com/report/sorting-for-circularity-india-wealth-in-waste/chapterdetail?reportid=813&chapter=1

24. Reverse Resources. *The Challenges of Textile Waste Management in India When Turning Fashion Circular* (Reverse Resources, 2020); https://reverseresources.net/news/the-challenges-of-textile-waste-management-in-india-when-turning-fashion-circular

25. Garvert, U. *A comparative study of recycling in the European and Brazilian textile industry* (Master thesis, 2017)

26. Sattar, S., Akhtar, N. *EU's Textile Waste and Used Clothing in Pakistan* (Global Village, 2023); https://www.globalvillagespace.com/eus-textile-waste-and-used-clothing-in-pakistan/

27. Garson & Shaw. *Exploring the Benefits of the Growing Used Textile Recycling Industry Located in the Karachi Export Processing Zone in Pakistan* (Garson & Shaw, 2019) https://www.garsonshaw.com/2019/11/exploring-the-benefits-of-the-growing-used-textile-recycling-industry-located-in-the-karachi-export-processing-zone-in-pakistan/

28. Daystar et al. (2019). Quantifying Apparel Consumer Use Behaviour in Six Countries: Addressing a Data Need in LCA Modeling. *J. Text. App. Tech. Manag.* **11** (2019)

29. Earth Action. *Plasteax database* (Earth Action, 2023); https://plasteax.earth/

30. Kaza, S., Yao, L., Bhada-Tata, P. & Van Woerdern, F. *What a Waste 2.0: A Global Snapshot of Solid Waste Management to 2050* (World Bank, 2018).

31. Watson et al. *Towards 2025: Separate Collection and Treatment of Textiles in Six EU Countries* (Danish Environmental Protection Agency, 2020)

32. Eurostat. *Waste Generation and Treatment Database, Number and Capacity of Recovery and Disposal Facilities by NUTS 2 Regions* (Eurostat, 2023); https://ec.europa.eu/eurostat/databrowser/view/env_wasfac/default/table?lang=en

33. European Commission. *Illegal Treatment and Disposal of Waste, Landfill Directive - Most recent CJEU Case Law* (European Commission, 2023); https://www.era-comm.eu/combatting_waste_crime/module_3/module_3_5.html

34. Gaulier, G., & Zignago, S.. Baci: *International Trade Database at the Product-Level.* (Centre d'Etudes Prospectives et d'Informations Internationales (CEPII), 2010)

35. Köhler et al. *Circular economy perspectives in the EU Textile sector* (Joint Research Center (JRC), 2021)

36. *Communication from local textile collectors and recyclers.*

37. PLASTEAX. *Mismanaged Waste Index (MWI) calculation, Methodology presentation* (Earth Action, 2021); https://www.plasteax.org/methodology.

38. WWF. *ReSource Footprint Tracker, Methodology Overview* (WWF), 2020).

39. Lebreton, L. C. M. et al. River plastic emissions to the world's oceans. *Nat. Commun.* **8**, 15611 (2017).

40. UN. *UN Comtrade Database* (UN, 2019); https://comtradeplus.un.org/
