# Supplementary material for: The global apparel industry is a significant yet overlooked source of plastic leakage
Source: Nat Commun. 2024 Jun 12;15:5022. doi: 10.1038/s41467-024-49441-4 (PMC11169549; doi:10.1038/s41467-024-49441-4)
Supplement: Supplementary file 3 — Description of Additional Supplementary Files [file 41467_2024_49441_MOESM3_ESM.pdf]

Description of Additional Supplementary Files

Supplementary Data 1:

Name	Description
Summary table	Results summary table
Graph waste	Graphs for plastic waste - focus on synthetic garments
Graph leakage	Graphs for plastic leakage - focus on synthetic garments
Results_mean	Leakage and waste quantity results per geography - average
Results_lower_end	Leakage and waste quantity results per geography - lower end
Results_higher_end	Leakage and waste quantity results per geography - higher end
Calculations_synthetic_mean	Calculations of leakage in the synthetic apparel value chain, per market - considering average MWI
Calculations_synthetic_lower_MWI	Calculations of leakage in the synthetic apparel value chain, per market - considering low MWI
Calculations_synthetic_higher_MW	Calculations of leakage in the synthetic apparel value chain, per market - considering high MWI
Calculation_cotton_mean	Calculations of leakage in the cotton apparel value chain, per market - considering average MWI
Calculation_cotton_lower_MWI	Calculations of leakage in the cotton apparel value chain, per market - considering low MWI
Calculation_cotton_higher_MWI	Calculations of leakage in the cotton apparel value chain, per market - considering high MWI
Calculation_other_fibers_mean	Calculations of leakage in the apparel value chain for other fibers, per market - considering average MWI
Calculation_other_fibers_lower	Calculations of leakage in the apparel value chain for other fibers, per market - considering low MWI
Calculation_other_fibers_higher	Calculations of leakage in the apparel value chain for other fibers, per market - considering high MWI
Average product	Generic consumption and leakage numbers per kg of product, for synthetic and cotton garments
Consumption Final Numbers	Summary of consumption numbers considered
Consumption Comparison	Apparel imports and consumption per market
Imports - EU	Imports of textiles in EU countries
Consumption MMF net Use	Consumption of Man-made fibers per market
Consumption Cotton net use	Consumption of cotton fibers per market
Consumption Cotton net use - EU	Consumption of cotton fibers per EU country
USDA loss factor	USDA loss facors applied on the apparel value chain
Consumption Data classification	USDA categories classification of textile products
Macro release	Macroplastic leakage rates per country, extracted from the Plastic Leak Project and Plasteax

Micro release	Microplastic leakage rates per country, extracted from the Plastic Leak Project
Plastic in Cotton cultivation	Calculations of plastic used on average in cotton supply chain
Used exports - EU high income	Exports of used textile per market - EU high income, data extracted from UN Comtrade
Used exports - EU low income	Exports of used textile per market - EU low income, data extracted from UN Comtrade
Used exports - all other markets	Exports of used textile per market - all except EU, data extracted from TDM
Used exports - TDM raw data	Extract of raw TDM data on exports of used textile
Manufact. Countries breakdown	Breakdown of volumes of imports per manufacturing location for US, EU and Japan sales markets, data extracted from TDM
Garment breakdown	Breakdown of volumes of imports per garment type, market and fiber type, data extracted from TDM
